# Association between gastroesophageal reflux disease and stroke: a bidirectional Mendelian randomization study

**DOI:** 10.3389/fneur.2023.1295051

**Published:** 2024-01-23

**Authors:** Decheng Meng, Xin Zhang, Wenfei Yu, Guoliang Yin, Suwen Chen, Hongshuai Liu, Linya Wang, Fengxia Zhang

**Affiliations:** ^1^First College of Clinical Medicine, Shandong University of Traditional Chinese Medicine, Jinan, China; ^2^College of Chinese Medicine, Shandong University of Traditional Chinese Medicine, Jinan, China; ^3^Department of Neurology, Affiliated Hospital of Shandong University of Traditional Chinese Medicine, Jinan, China

**Keywords:** stroke, gastroesophageal reflux disease, Mendelian randomization, causality, single-nucleotide polymorphisms

## Abstract

**Objective:**

Some previous studies have suggested a potential link between stroke and gastroesophageal reflux disease (GERD). We used a two-sample bidirectional Mendelian randomization (MR) method to explore the causal relationship between stroke and GERD.

**Design:**

Summary-level data derived from the published genome-wide association studies (GWAS) were employed for analyses. Single-nucleotide polymorphisms (SNPs) as instrumental variables (IVs) for stroke (*n* = 446,696) and its common subtypes ischemic stroke (IS) (*n* = 440,328), large vessel stroke (LVS) (*n* = 410,484), small vessel stroke (SVS) (*n* = 198,048), and cardioembolic stroke (CES) (*n* = 413,304) were obtained from the MEGASTROKE consortium. The data on intracerebral hemorrhage (ICH) (*n* = 721,135) come from the UK Biobank. Instrumental variables (IVs) for lacunar stroke (LS) (*n* = 474,348) and GERD (*n* = 602,604) were screened from publicly available genetic summary data. The inverse variance weighted (IVW) method was used as the main MR method. Pleiotropy was detected by the MR-Egger intercept test, MR pleiotropy residual sum and outlier, and leave-one-out analysis. Cochran Q statistics were used as supplements to detect pleiotropy.

**Results:**

We found that GERD can causally increase the risk of stroke [IVW odds ratio (OR): 1.22, 95% confidence interval (CI): 1.13–1.32, *p* = 1.16 × 10^−6^] and its common subtypes IS (OR: 1.19, 95% CI: 1.10–1.30, *p* = 3.22 × 10^−5^), LVS (OR: 1.49, 95% CI: 1.21–1.84, *p* = 1.47 × 10^−4^), and LS (OR: 1.20, 95% CI: 1.001–1.44, *p* = 0.048). Several important risk factors for stroke have also been implicated in the above causal relationship, including type 2 diabetes, sleep apnea syndrome, high body mass index, high waist-to-hip ratio, and elevated serum triglyceride levels. In reverse MR analysis, we found that overall stroke (OR: 1.09, 95% CI: 1.004–1.19, *p* = 0.039) and IS (OR: 1.10, 95% CI: 1.03–1.17, *p* = 0.007) have the causal potential to enhance GERD risk.

**Conclusion:**

This MR study provides evidence supporting a causal relationship between GERD and stroke and some of its common subtypes. We need to further explore the interconnected mechanisms between these two common diseases to better prevent and treat them.

## 1 Introduction

Gastroesophageal reflux disease (GERD) and stroke are two common medical clinical diseases, and their incidence and related health and economic burdens have been increasing in recent years. Stroke is a common cerebrovascular disease caused by neurological deficits associated with acute focal injury to the central nervous system. According to its pathological mechanisms, stroke can be divided into hemorrhagic stroke and ischemic stroke. Ischemic stroke due to blocked arteries and decreased blood flow to the brain accounts for the majority of stroke patients. In recent years, the incidence of stroke, especially ischemic stroke, has gradually increased and has become one of the important causes of death and disability worldwide, causing a severe socioeconomic burden (1). GERD is a digestive system disease that causes various esophageal lesions and related complications due to the reflux of gastric contents (2). The global incidence of gastroesophageal reflux has increased significantly in the past 20 years, and its prevalence in North America (18.1–27.8%) and Europe (8.8–25.9%) is significantly higher than that in East Asia (2.5–7.8%) (3).

Some past studies have found that nearly 50% of stroke patients will develop gastrointestinal complications such as dysphagia and gastrointestinal bleeding after the disease (4). In addition, because of the close connection between the esophagus and the left atrium, GERD is thought to have an intimate role in atrial fibrillation, and some studies have linked GERD to an increased risk of atrial fibrillation, which increases the risk of stroke (5). In recent years, some studies have further suggested that there may be a close relationship between stroke and GERD. A population-based cohort study found that stroke patients had a higher incidence (1.51 times) of gastroesophageal reflux disease than non-stroke subjects (6). A population-based follow-up study suggests that young patients with gastric reflux esophagitis are at higher risk of stroke (7). An analysis of inpatient samples also found that reflux esophagitis increased the incidence of acute stroke in patients with atrial fibrillation (8). It has long been considered that there may be a link between GERD and stroke. However, the causal relationship between GERD and stroke and its subtypes has not been confirmed. Suppose the possible potential causal relationship between GERD and stroke can be clarified, in that case, it will be of great significance to prevent and treat these two common medical clinical diseases.

Mendelian randomization (MR) is an analytical method that uses genetic variants as instrumental variables (IVs) for test exposures to assess the causal role of their associations with clinical outcomes. The application of MR avoids unobserved confounding factors and reverse causation in evaluating the relationship between exposure and outcome, thus becoming a valuable medical research tool (9). We compiled some GWAS data and used MR for the first time to evaluate whether there is a bidirectional causal relationship between stroke and its subtypes and gastroesophageal reflux disease. Based on the two-sample, two-way MR method, we studied the potential bidirectional causal relationship between stroke and its common subtypes, ischemic stroke (IS), large vessel stroke (LVS), small vessel stroke (SVS), cardioembolic stroke (CES), lacunar stroke (LS), intracranial hemorrhage (ICH), and GERD.

## 2 Materials and methods

### 2.1 Study design

**Figure 1:**
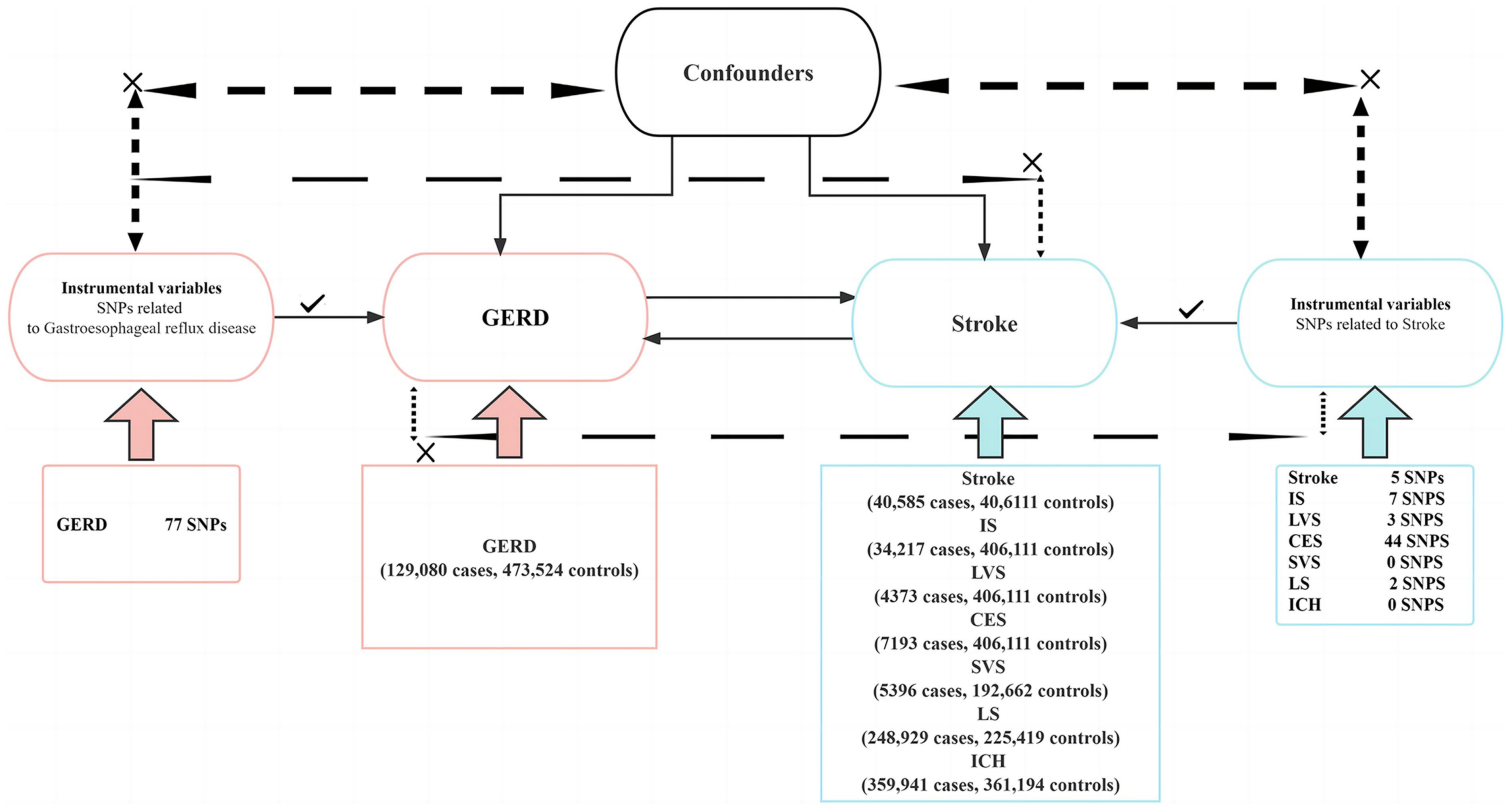
This is a two-sample bidirectional Mendelian randomization experimental design. SNPs, single-nucleotide polymorphisms; GERD, gastroesophageal reflux disease; IS, ischemic stroke; LVS, large vessel stroke; CES, cardioembolic stroke; SVS, small vessel stroke; LS, lacunar stroke; ICH, intracranial hemorrhage.

We performed a two-sample bidirectional MR study using single-nucleotide polymorphisms (SNPs) as IVs to investigate the causal relationship between stroke and its common subtypes (IS, LVS, SVS, CS, LS, and ICH) and GERD. The workflow of this Mendelian randomization is shown in Figure 1. The design and analysis of MR must be based on the following three key assumptions: (1) correlation: SNPs used as IVs must be associated with exposure; (2) exclusivity: IVs can only affect outcomes through exposure; and (3) independence: IVs cannot influence results through any unmeasured confounding variables. The data used in this MR study are all public data from published studies, so ethical approval and informed consent are not required. The data statistics in this study were conducted using the TwoSampleMR software package and the MR PRESSO software package in R (version 4.3.1). The study design and reporting conformed to using STROBE-MR (10).

### 2.2 Data sources and SNP selection for stroke

The summary-level data for stroke and its common subtypes IS, LVS, CES, and SVS were obtained from genome-wide association studies (GWAS) conducted by the MEGASTROKE consortium. The MEGASTROKE consortium performed a meta-analysis of 29 GWAS involving 520,000 European ancestry participants (including 40,5,850 stroke patients and 406,111 controls) and identified a total of 32 stroke risk loci (including 10 previously discovered loci and 22 new loci) (11). The GWAS data of lacunar stroke come from a recent meta-analysis conducted by International Stroke Genetics Consortium (ISGC), including 225,419 subjects of European ancestry (including 6,030 LS patients and 248,929 controls) (12). The summary data for ICH was extracted from the UK Biobank (359,941 cases and 361,194 controls). By excluding SNPs with *p* > 5 × 10^−8^ and *r*^2^ > 0.01, we extracted 8 SNPs, 9 SNPs, 4 SNPs, 47 SNPs, 3 SNPs, and 1 SNP from the GWAS data of overall stroke, IS, LVS, CES, LS, and ICH, respectively. After that, by removing weak IVs with an F statistic less than 10 and instrumental variables containing confounding factors (by consulting http://www.phenoscanner.medschl.http://cam.ac.uk/), we finally retained 5 SNPs of stroke, 7 SNPs of IS, 3 SNPs of LVS, 3 SNPs of CES, and 2 SNPs of LS. However, there was a lack of IVs of SVS and ICH after excluding them. More details about the trait definition are available in Supplementary Tables S8–S12.

### 2.3 Data sources and SNP selection for gastroesophageal reflux disease

The summary data for GERD phenotypes were obtained from a multi-trait genetic association analysis of GERD. This analysis identified 88 loci associated with GERD by including 129,080 cases and 473,524 controls with a total of 602,604 subjects (13). By excluding SNPs with *p* > 5 × 10^−8^ and *r*^2^ > 0.01, we extracted 80 SNPs from GWAS data of GERD. By removing weak IVs (*F* < 10) and instrumental variables containing confounding factors, we finally retained 77 SNPs. More details about the trait definition are available in Supplementary Tables S1–S7. The GWAS data used in this study are detailed in Table 1.

**Table 1:** Details of the GWAS databases involved in Mendelian randomization.

Phenotype	Consortium	Participants (cases/controls)	Ancestry	PubMed ID
Gastroesophageal reflux disease	NA	129,080/473,524	European	34,187,846
Stroke	MEGASTROKE	40,585/406,111	European	29,531,354
Ischemic stroke	MEGASTROKE	34,217/406,111	European	29,531,354
Large vessel stroke	MEGASTROKE	4,373/406,111	European	29,531,354
Cardioembolic stroke	MEGASTROKE	7193/406,111	European	29,531,354
Small vessel stroke	MEGASTROKE	5,386/192,662	European	29,531,354
Lacunar stroke	NA	248,929/225,419	European	33,773,637
Intracranial hemorrhage	UK Biobank	359,941/361,194	European	

Data summarized from GWAS databases from IEU OpenGWAS database (https://gwas.mrcieu.ac.uk/).

### 2.4 Statistical analysis

In this study, we adopted inverse-variance weighting (IVW) as the preferred method and conducted a two-sample bidirectional MR analysis to explore the causal relationship between stroke and GERD. Although the IVW method has high statistical power, this method may also treat some IVs that affect the results through other pathways as valid SNPs (14). Therefore, to avoid bias in causality assessment, we used MR-Egger regression, weighted median, weighted mode, and MR pleiotropy residual sum and outlier (MR-PRESSO) approaches for further sensitivity analysis. MR-Egger regression can assess potential horizontal pleiotropy, and MR-PRESSO approaches can identify and remove these pleiotropic outliers (15). The weighted median method enables consistent causal estimation when more than half of the IVs are valid (16). Weighted mode can evaluate the causal relationship between the results and a subset of SNPs with similar causal effects (17). Finally, we also used the leave-one-out method to assess whether omitting a specific SNP would significantly impact the results of MR.

### 2.5 Risk factor

To explore the underlying genetic mechanisms of GERD combined with stroke and stroke combined with GERD, we also analyzed the relationship between GERD and stroke-related risk factors and the relationship between stroke and GERD-related risk factors by referring to the study published by Tao H et al. (18). Based on relevant research data, we included stroke-related risk factors such as hypertension, hyperlipidemia (HDL cholesterol and LDL cholesterol), hypertriglyceridemia (triglyceride), type 2 diabetes, sleep apnea syndrome, and obesity (body mass index and waist–hip ratio) (19). Research shows risk factors for gastroesophageal reflux, including age, smoking, NSAID use, and obesity (20). Therefore, we explored the relationship between stroke and obesity (body mass index and waist–hip ratio). The GWAS data for these risk factors are all from the UK Biobank. The details of each data source are presented in Table 2. We performed MR using GERD or stroke as exposures and the above risk factors as outcome factors. IVW was used as the main analysis method, and a value of *p* of <0.05 was considered significant.

**Table 2:** GWAS database details of risk factors involved in stroke and gastroesophageal reflux disease.

Phenotype	Consortium	Participants (cases/controls)	Ancestry	PubMed ID
Hypertension	UK Biobank	129,909/354,689	European	33,959,723
HDL cholesterol	UK Biobank	403,943	European	32,203,549
LDL cholesterol	UK Biobank	440,546	European	32,203,549
Triglyceride	UK Biobank	441,016	European	32,203,549
Type 2 diabetes	UK Biobank	3,8,841/451,248	European	34,594,039
Sleep apnea syndrome	UK Biobank	13,818/463,035	European	34,594,039
Body mass index	UK Biobank	532,396	European	29,892,013
Waist-hip ratio	UK Biobank	502,773	European	29,892,013

Data summarized from GWAS databases from IEU OpenGWAS database (https://gwas.mrcieu.ac.uk/).

HDL cholesterol, high-density lipoprotein cholesterol; LDL cholesterol, low-density lipoprotein cholesterol.

## 3 Results

### 3.1 The causal effect of gastroesophageal reflux disease on stroke

As revealed by the MR analyses, there was a significant correlation between GERD and increased risk of overall stroke [IVW odds ratio (OR): 1.22, 95% confidence interval (CI): 1.13–1.32, *p* = 1.16 × 10^−6^], IS (OR: 1.19, 95% CI: 1.10–1.30, *p* = 3.22 × 10^−5^), LVS (OR: 1.49, 95% CI: 1.21–1.84, *p* = 1.47 × 10^−4^), and LS (OR: 1.20, 95% CI: 1.001–1.44, *p* = 0.048). However, IVW method analysis showed no causal relationship between GRED and CES (OR: 1.04, 95% CI: 0.90–1.21, *p* = 0.563), and ICH (OR: 1.00, 95% CI: 1.00–1.00, *p* = 0.215). The results of other MR methods (including weighted median, MR-Egger, simple mode, and weighted mode) analyzing the relationship between GERD and overall stroke, IS, LVS, and LSs were consistent with IVW. MR results assessing the causal impact of GERD on the risk of stroke and its common subtypes are shown in Table 3.

**Table 3:** MR analysis results of various methods used to assess the causal relationship between GERD and stroke and its common subtypes.

Outcome	No. of SNPs	Methods	*p*	OR (95% CI)
Stroke	77	MR Egger	0.783	1.07 (0.67, 1.69)
		Weighted median	<0.001	1.22 (1.10, 1.36)
		Inverse variance weighted	<0.001	1.22 (1.13, 1.32)
		Simple mode	0.039	1.33 (1.02, 1.75)
		Weighted mode	0.053	1.30 (1.01, 1.68)
Ischemic stroke	77	MR Egger	0.756	1.08 (0.67, 1.75)
		Weighted median	0.001	1.20 (1.08, 1.34)
		Inverse variance weighted	<0.001	1.19 (1.10, 1.30)
		Simple mode	0.155	1.25 (0.92, 1.69)
		Weighted mode	0.116	1.23 (0.95, 1.58)
Large vessel stroke	76	MR Egger	0.546	1.44 (0.44, 4.67)
		Weighted median	0.021	1.37 (1.05, 1.79)
		Inverse variance weighted	<0.001	1.49 (1.21, 1.84)
		Simple mode	0.318	1.37 (0.74, 2.55)
		Weighted mode	0.210	1.41 (0.83, 2.42)
Cardioembolic stroke	76	MR Egger	0.311	0.64 (0.28, 1.50)
		Weighted median	0.580	1.06 (0.87, 1.28)
		Inverse variance weighted	0.563	1.04 (0.90, 1.21)
		Simple mode	0.552	1.16 (0.72, 1.87)
		Weighted mode	0.791	1.06 (0.67, 1.69)
Small vessel stroke	77	MR Egger	0.625	1.32 (0.43, 4.05)
		Weighted median	0.501	1.30 (0.99, 1.68)
		Inverse variance weighted	0.174	1.15 (0.94, 1.39)
		Simple mode	0.236	1.43 (0.79, 2.58)
		Weighted mode	0.253	1.40 (0.79, 2.46)
Lacunar stroke	65	MR Egger	0.604	1.31 (0.47, 3.64)
		Weighted median	0.253	1.16 (0.90, 1.49)
		Inverse variance weighted	0.048	1.20 (1.001, 1.44)
		Simple mode	0.916	1.03 (0.55, 1.94)
		Weighted mode	0.761	1.09 (0.62, 1.92)
Intracranial hemorrhage	77	MR Egger	0.315	1.00 (0.99, 1.00)
		Weighted median	0.624	1.00 (1.00, 1.00)
		Inverse variance weighted	0.215	1.00 (1.00, 1.00)
		Simple mode	0.960	1.00 (1.00, 1.00)
		Weighted mode	0.976	1.00 (1.00, 1.00)

**Figure 2:**
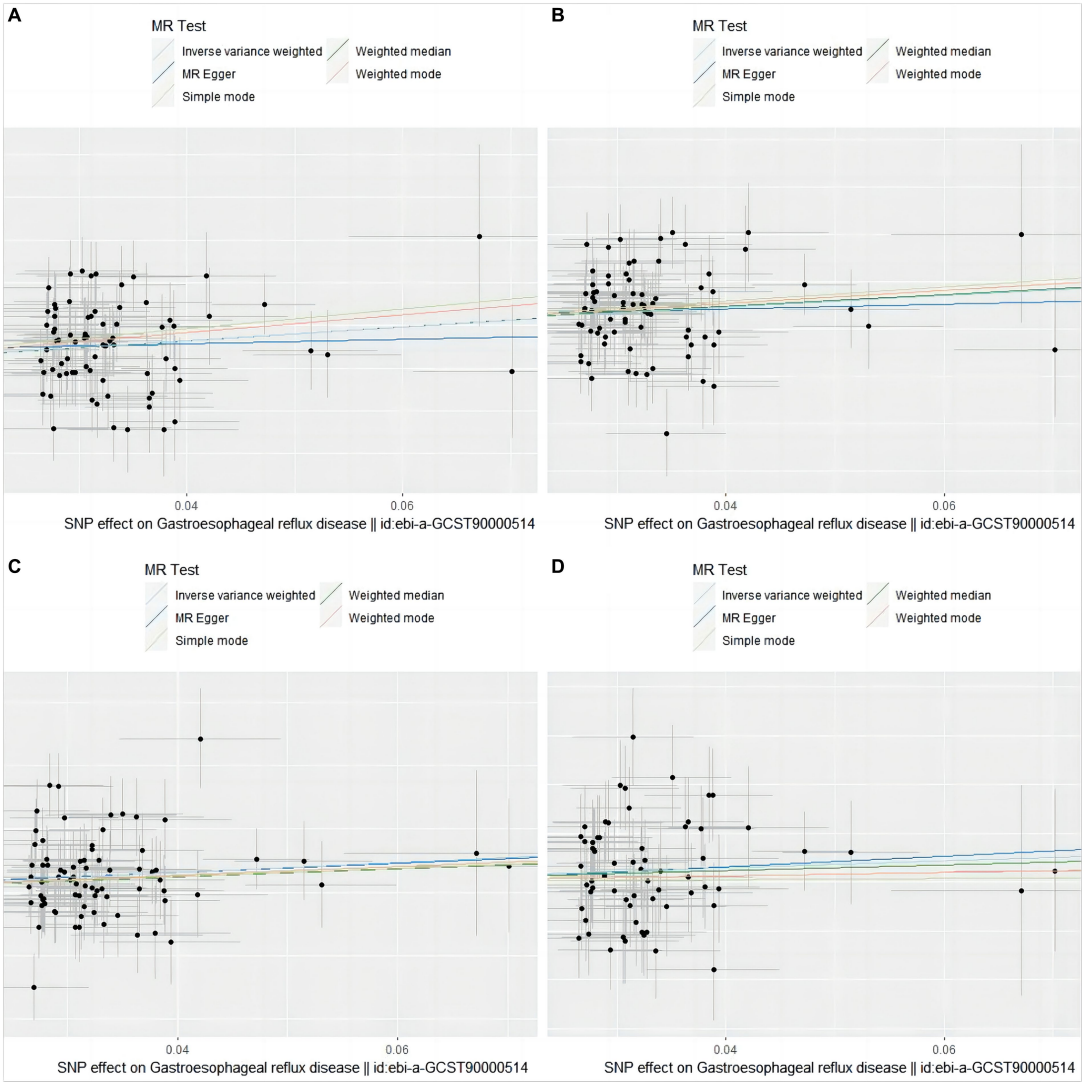
Scatter plot of genetic association between GERD and overall stroke, IS, LVS, and LS. **(A)** stroke; **(B)** ischemic stroke; **(C)** large vessel stroke; **(D)** lacunar stroke. The slope and direction of the straight line represent the magnitude and direction of the causal relationship.

The causal relationship between GERD and overall stroke risk, IS, LVS, and LS can be visually displayed through scatter plots (Figure 2) and forest plots (Supplementary Figure S1).

The MR Egger intercept test found no pleiotropic effect (all p-val > 0.05). The MR PRESSO test of GERD for IS, LVS, CES, SVS, LS, and ICH also did not detect a pleiotropic effect (p-val > 0.05). The Cochran Q test of GERD for IS, LVS, CES, SVS, LS, and ICH did not detect heterogeneity (Q_p-val > 0.05). The Cochran Q test only detected heterogeneity in the overall stroke (Q_p-val = 0.020) (Table 4).

**Table 4:** Heterogeneity and horizontal pleiotropy analyses between GERD and stroke and its common subtypes.

	Pleiotropy test				Heterogeneity test					
	MR_Egger			MR_presso	MR_Egger			IVW		
Outcome	Intercept	SE	*p* value	*p* value	Q	Q_*d*f	Q_*p* value	Q	Q_*d*f	Q_*p* value
Stroke	0.004	0.008	0.564	0.034	102.109	75	0.020	102.565	76	0.023
Ischemic stroke	0.003	0.008	0.677	0.105	93.855	75	0.069	94.075	76	0.078
Large vessel stroke	0.001	0.020	0.950	0.094	91.037	74	0.087	91.042	75	0.100
Cardioembolic stroke	0.016	0.014	0.260	0.066	86.762	74	0.147	88.274	75	0.140
Small vessel stroke	−0.005	0.019	0.797	0.087	96.221	75	0.050	96.306	76	0.058
Lacunar stroke	−0.003	0.017	0.861	0.243	71.855	63	0.208	71.890	64	0.233
Intracranial hemorrhage	<0.001	<0.001	0.214	0.112	92.324	75	0.085	94.260	76	0.076

**Figure 3:**
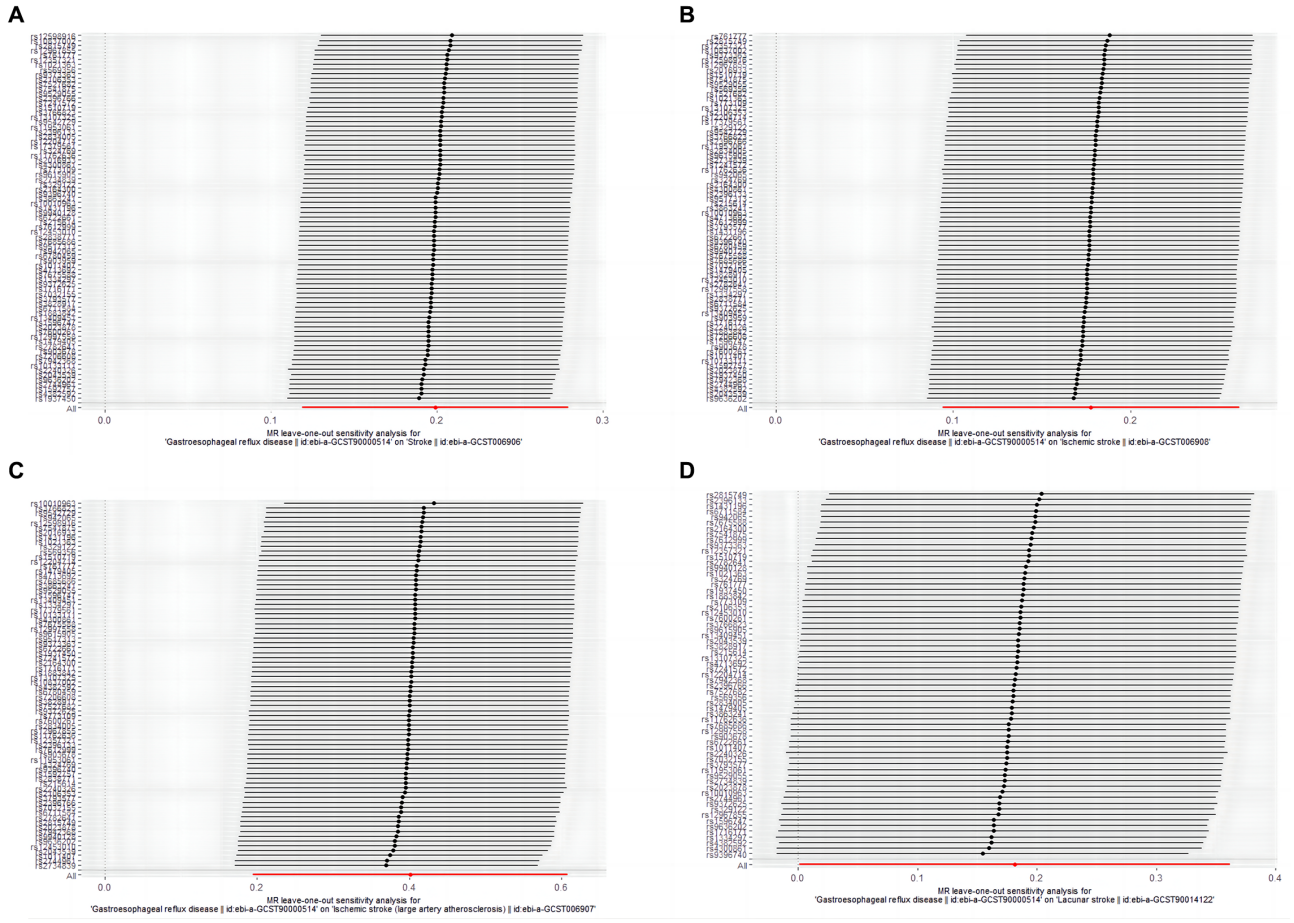
The leave-one-out-sensitivity forest plot of genetic association between GERD and overall stroke, IS, LVS, and LS. **(A)** stroke; **(B)** ischemic stroke; **(C)** large vessel stroke; **(D)** lacunar stroke.

In the leave-one-out analysis, no single SNP of GERD with a significant effect on MR results was present (Figure 3). Funnel plots (Supplementary Figure S2) also show the distribution of GERD on the risk of stroke, and its common subtypes were roughly symmetrical when a single SNP was used as an IV.

### 3.2 The causal effect of stroke on gastroesophageal reflux disease

The IVW result showed that overall stroke (OR: 1.09, 95% CI: 1.004–1.19, *p* = 0.039) and IS (OR: 1.10, 95% CI: 1.03–1.17, *p* = 0.007) have an apparent causal effect on the increased risk of GERD. Other complementary MR methods also validated the results obtained from the IVW analysis. However, the results of MR analysis showed no significant causal relationship among LVS (OR: 1.01, 95% CI: 0.96–1.07, *p* = 0.609), CES (OR: 1.02, 95% CI: 0.98–1.05, *p* = 0.320), LS (OR: 1.04, 95% CI: 0.99–1.10, *p* = 0.141), and GERD. Because SVS and ICH lacked sufficient SNPs to be used as IVs, their causal relationship with GERD risk could not be assessed. The MR results evaluating the causal impact of stroke and its common subtypes on the risk of GERD are shown in Table 5.

**Table 5:** MR analysis results of various methods used to assess the causal relationship between stroke and its common subtypes and GERD.

Exposure	No. of SNPs	Methods	*p*	OR (95% CI)
Stroke	5	MR Egger	0.945	1.02 (0.59, 1.75)
		Weighted median	0.116	1.08 (0.98, 1.19)
		Inverse variance weighted	0.039	1.09 (1.004, 1.19)
		Simple mode	0.233	1.11 (0.96, 1.29)
		Weighted mode	0.279	1.09 (0.95, 1.25)
Ischemic stroke	7	MR Egger	0.426	1.26 (0.75, 2.14)
		Weighted median	0.053	1.08 (1.01, 1.17)
		Inverse variance weighted	0.007	1.10 (1.03, 1.17)
		Simple mode	0.299	1.08 (0.95, 1.23)
		Weighted mode	0.306	1.08 (0.94, 1.23)
Large vessel stroke	3	MR Egger	0.281	1.42 (1.03, 1.97)
		Weighted median	0.643	1.01 (0.96, 1.06)
		Inverse variance weighted	0.609	1.01 (0.96, 1.07)
		Simple mode	0.879	0.99 (0.92, 1.07)
		Weighted mode	0.629	1.02 (0.95, 1.10)
Cardioembolic stroke	3	MR Egger	0.489	1.05 (0.96, 1.14)
		Weighted median	0.337	1.02 (0.98, 1.05)
		Inverse variance weighted	0.320	1.02 (0.98, 1.05)
		Simple mode	0.971	0.99 (0.95, 1.05)
		Weighted mode	0.333	1.03 (0.99, 1.07)
Lacunar stroke	2	MR Egger		
		Weighted median		
		Inverse variance weighted	0.141	1.04 (0.99, 1.10)
		Simple mode		
		Weighted mode		

The causal relationship between overall stroke, IS, and the risk of GERD can be visually displayed through scatter plots (Supplementary Figure S3) and forest plots (Supplementary Figure S4).

Cochran Q and MR Egger intercept tests found no heterogeneity or directional pleiotropy between overall stroke, IS, LVS, CES, and GERD (*p*-val > 0.05). The MR PRESSO test also found no evidence of a pleiotropic effect between overall stroke, IS, CES, and GERD (*p*-val > 0.05). Although the MR Egger intercept test of causality between LS and GERD found no heterogeneity (p-val > 0.05), the Cochran Q test could not be performed due to the lack of sufficient available SNPs (Table 6).

**Table 6:** Heterogeneity and horizontal pleiotropy analyses between stroke and its common subtypes and GERD.

	Pleiotropy test				Heterogeneity test					
	MR_Egger			MR_PRESSO	MR_Egger			IVW		
Exposure	Intercept	SE	*p* val	*p* val	Q	Q_*p*f	Q_*p* value	Q	Q_*d*f	Q_*p* value
Stroke	0.005	0.019	0.821	0.344	5.035	3	0.169	5.137	4	0.273
Ischemic stroke	−0.011	0.021	0.617	0.237	8.012	5	0.156	8.467	6	0.206
Large vessel stroke	−0.070	0.034	0.289		<0.001	1	0.988	4.194	2	0.123
Cardioembolic stroke	−0.006	0.008	0.611	0.669	0.113	1	0.736	0.603	2	0.740
Lacunar stroke	NA	NA	NA		NA	NA	NA	0.181	1	0.670

In the leave-one-out analysis, no single SNP with a significant effect on MR results was present (Supplementary Figure S5).

### 3.3 Risk factor analysis

To further investigate potential mediators of increased risk of GERD and stroke, we used MR analysis to evaluate the causal relationship between GERD on common risk factors for stroke and stroke on the risk factor of obesity for GERD. As shown in Table 7, GERD is causally linked to higher triglyceride levels (OR: 1.20, 95% CI: 1.17–1.23, *p* = 8.30 × 10^−41^), type 2 diabetes (OR: 1.58, 95% CI: 1.41–1.78, *p* = 4.80 × 10^−5^), higher BMI (OR: 1.44, 95% CI: 1.35–1.54, *p* = 8.63 × 10^−9^), greater waist-to-hip ratio (OR: 1.05, 95% CI: 1.02–1.10, *p* = 0.001), and sleep apnea syndrome (OR: 1.75, 95% CI: 1.54–2.00, *p* = 3.46 × 10^−17^). Although there may also be a causal relationship between GERD and lower HDL cholesterol levels (OR: 0.81, 95% CI: 0.77–0.85, *p* = 8.42 × 10^−19^) and hypertension (OR: 1.03, 95% CI: 1.01–1.04, *p* = 0.0004), the MR Egger test of the causal relationship between them found heterogeneity (*p*-val<0.05). However, no causal relationship was found among stroke, higher BMI, and greater waist-to-hip ratio. According to risk factor analysis, hypertriglyceridemia, type 2 diabetes, obesity, and sleep apnea syndrome may be the reasons for the further increased risk of stroke caused by GERD.

**Table 7:** Result of the risk factors analysis.

		Inverse variance weighted		MR_Egger test		
Exposure	Outcome	*p*	OR (95% CI)	Intercept	SE	*p* value
GERD	Hypertension	<0.001	1.03 (1.01,1.04)	0.004	0.001	0.004
GERD	Triglycerides	<0.001	1.20 (1.17,1.23)	−0.003	0.003	0.249
GERD	HDLcholesterol	<0.001	0.81 (0.77,0.85)	0.017	0.004	<0.001
GERD	LDLcholesterol	0.081	0.98 (0.95,1.01)	0.007	0.002	0.004
GERD	T2DM	<0.001	1.58 (1.41,1.78)	−0.020	0.011	0.085
GERD	BMI	<0.001	1.44 (1.35,1.54)	−0.007	0.006	0.300
GERD	Waist-hip ratio	0.001	1.05 (1.02,1.10)	0.005	0.003	0.106
GERD	SAS	<0.001	1.75 (1.54,2.00)	−0.008	0.013	0.546
Stroke	BMI	0.728	0.99 (0.94,1.04)	−0.015	0.012	0.254
Stroke	Waist-hip ratio	0.279	1.03 (0.98,1.04)	0.005	0.014	0.764

HDL cholesterol, high-density lipoprotein cholesterol; LDL cholesterol, low-density lipoprotein. Cholesterol; T2DM, diabetes mellitus type 2; BMI, body mass index; SAS, sleep apnea syndrome.

## 4 Discussion

In recent years, as the incidence of GERD and stroke has continued to increase, there has been an increasing overlap between patients with these two common medical clinical diseases. Therefore, further exploration of the potential relationship between the two diseases is increasingly important to prevent and treat them. Several clinical observational studies in recent years have shown that GERD can increase the risk of stroke in different groups of people, such as regular GERD patients, young adult GERD patients, and GERD patients with atrial fibrillation (7, 8, 21). Similarly, a population-based cohort study also found that stroke patients had a higher risk of GERD (6). These studies may show a possible bidirectional correlation between GERD and stroke.

A previous MR study found a causal relationship between GERD and some cardiovascular diseases (CVD) (22). Although this study provides a detailed assessment of the causal relationship between GERD and multiple CVDs, including any stroke, IS and ICH, and numerous CVD risk factors, it was not able to further analyze the causal relationship between GERD and various common subtypes of IS, and the causal association between stroke and its subtypes and GERD risk. Another previous MR study evaluated the causal relationship between stroke and gastrointestinal diseases, including GERD (23). This study found that ICH, especially deep ICH, can causally increase the risk of gastrointestinal disorders such as GERD. This study also found a causal relationship between IS and GERD, but they failed to find a causal relationship between overall stroke and subtypes of IS and GERD. We first used the two-sample MR analysis of bidirectional causality to evaluate the causal relationship between GERD and stroke and its common subtypes (including IS, LVS, SVS, CES, LS, and ICH). Our research results are the first to demonstrate a bidirectional causal relationship between GERD and overall stroke, IS. In addition, GERD will also causally increase the risk of stroke common subtypes such as LVS and LS. However, our study did not find evidence of a causal association between GERD and CES, SVS, and ICH.

The cause of GERD leading to stroke and its common subtypes are not yet precise but may be related to vagal nerve dysfunction. GERD can cause an increased risk of vagal nervous system dysfunction by affecting regular esophageal transport, peristalsis, and normal function of the lower esophageal sphincter (24). Vagal nervous system dysfunction leads to impairment of the cholinergic anti-inflammatory pathway, leading to immune system dysregulation, increased inflammatory mediators in the bloodstream, and exaggerated immune response. The development and progression of atherosclerosis caused by excessive inflammatory response in blood vessels and impairment of cerebrovascular autonomic flow regulation function lead to an increased risk of strokes such as large vessel strokes (25, 26). In addition, studies have shown that esophageal acid reflux can not only cause esophageal peristalsis disorder and vagus nerve damage but also lead to a decrease in coronary blood flow, induce the occurrence of cardiovascular diseases such as angina, and thus increase the risk of cardiogenic stroke (27).

The causal relationship between stroke and the risk of GERD may be attributed to impaired voluntary control of oropharyngeal movements, changes in intestinal mucosal microbiota, and disruption of the gut–brain axis after brain injury. First, dysphagia after IS decreased esophageal sphincter muscle tone, and impairment of normal gastrointestinal motility function are important risk factors leading to the occurrence and aggravation of GERD. In addition, stroke can also exacerbate gastrointestinal symptoms such as dysphagia by affecting nutritional factors and neural centers regulating intestinal motility and appetite, such as the sympathetic and parasympathetic medulla and hypothalamic center, thereby further increasing the risk of GERD (4). Second, recent studies have found that the normal microbiota of the intestinal mucosa of mice is destroyed after brain injury caused by stroke. The main manifestations are the decrease in the level of Prevotellaceae, the increase in the abundance of Clostridium species, and the increase in the level of Myxobacteria (28, 29). A recent study pointed out that the imbalance of intestinal flora is involved in the occurrence and development of GERD (30). Therefore, the destruction of intestinal microbiota by stroke may be one of the critical factors leading to the increased risk of GERD. In addition, ischemic stroke can also lead to dysregulation of immune signal transduction and induce the development of brain–gut complications, such as gut microbiota dysbiosis, thereby further disrupting the normal circulation of the brain–gut axis, leading to the development of gastrointestinal diseases (31). Moreover, the causal relationship between GERD and stroke may also be related to common risk factors such as diet, smoking, and metabolic syndrome (obesity, hypertension, etc.) (32, 33). After we analyzed some related risk factors for stroke and GERD, it was found that GERD is causally responsible for the increased risk of stroke risk factors such as hypertriglyceridemia, type 2 diabetes, obesity, and sleep apnea syndrome.

Stroke and GERD are two common clinical diseases that cause substantial health risks and economic burdens worldwide. Since there is a proven bidirectional causal relationship between the two diseases, clinicians need to guide GERD patients to prevent the occurrence of stroke, and stroke patients should also pay attention to the potential risk of GERD. A population-based study points to a bidirectional causal relationship between GERD and sleep problems (34). Another recent study showed that insomnia increases the risk of stroke by following 31,126 participants for 9 years (35). Similarly, one study analysis found a bidirectional association between GERD and depression (36). Another prospective study found that major depressive episodes significantly increase the risk of stroke (37). Therefore, improving sleep and mood problems in GERD patients may effectively reduce the risk of stroke.

For patients at potential risk of stroke or those following a stroke, the use of drugs such as aspirin, calcium channel blockers, or the use of some non-oral feeding methods such as nasogastric (NG) tubes may lead to a higher risk of stroke. Aspirin is a commonly used drug to prevent ischemic stroke in patients who have experienced a cardiovascular event such as stroke or have risk factors for stroke such as atherosclerosis and antiphospholipid antibody syndrome (38). However, research suggests taking aspirin may lead to an increased risk of gastrointestinal diseases such as GERD. A study shows that long-term use of PA32540 (enteric-coated aspirin 325 mg + immediate-release omeprazole 40 mg) can effectively reduce the risk of gastrointestinal diseases caused by aspirin alone (39). This discovery may provide a new and better medication regimen for patients who use aspirin to prevent the occurrence or recurrence of stroke in the future. In addition, when treating hypertension, a significant risk factor for stroke, attention should also be paid to the gastrointestinal risks caused by antihypertensive drugs such as calcium antagonists (40). Furthermore, for patients who require non-oral feeding methods due to dysphagia after stroke, research has found that compared with NG tube feeding, esophageal (OE) tubes may lead to a smaller risk of developing and developing GERD (41). This discovery may help clinicians better select appropriate non-oral feeding methods for stroke patients.

Our study relied on the two-sample bidirectional MR analysis method to explore the causal relationship between stroke and its common subtypes and GERD. Compared with traditional research, on the one hand, our research effectively enhances the reliability of the research results by avoiding the influence of confounding factors and reverse causality. On the other hand, the instrumental variables selected in our study come from rigorously screened GWAS summary data based on individuals of European ancestry and effectively avoid the adverse effects of population stratification and instrumental variable bias on research results. In addition, the GWAS data on GERD, stroke and its subtypes, and related risk factors we selected are all from different consortiums, and the ancestry of these data is European, so we can rule out the possibility of a high overlap rate of exposure and outcome cohorts.

Honestly, our research still has some flaws: because our data were derived from GWAS data pooled from other studies, the sex and age of each sample were not known, and there may have been overlap in IVs used for exposure and outcomes. Since the samples are all from people of European ancestry, the study’s results may not apply to people of ancestry from other parts of the world, such as East Asia or North America. Our study only found a bidirectional causal relationship among overall stroke, IS, and GERD, so unfortunately, we cannot determine which specific subtypes of stroke can causally increase the risk of GERD. Additionally, due to the lack of available IVs for SVS and ICH, we cannot assess the causal relationship between SVS, ICH, and GERD. These deficiencies may be the focus of future research using MR or other research methods to explore the relationship between stroke and GERD.

## 5 Conclusion

In summary, results from this MR study provide evidence for the existence of a causal relationship between GERD and stroke and its common subtypes. The specific mechanism of the causal relationship between the two still needs to be further explored in the future to better guide the diagnosis and treatment of stroke, GERD, and their complications.

## Data availability statement

The original contributions presented in the study are included in the article/Supplementary material, further inquiries can be directed to the corresponding author.

## Ethics statement

Ethical review and approval was not required for the study on human participants in accordance with the local legislation and institutional requirements. Written informed consent from the patients/participants or patients/participants’ legal guardian/next of kin was not required to participate in this study in accordance with the national legislation and the institutional requirements.

## Author contributions

DM: Conceptualization, Data curation, Investigation, Methodology, Software, Writing – original draft, Writing – review & editing. XZ: Validation, Writing – review & editing. WY: Validation, Writing – review & editing. GY: Validation, Writing – review & editing. SC: Validation, Writing – review & editing. HL: Supervision, Writing – review & editing. LW: Supervision, Writing – review & editing. FZ: Writing – review & editing.
