# Supplementary material for: Association between gastroesophageal reflux disease and stroke: a bidirectional Mendelian randomization study
Source: Front Neurol. 2024 Jan 23;14:1295051. doi: 10.3389/fneur.2023.1295051 (PMC10844487; doi:10.3389/fneur.2023.1295051)
Supplement: Supplementary file 1 [file Data_Sheet_1.docx]

Supplementary Material

Association between Gastroesophageal reflux disease and Stroke: A bidirectional Mendelian randomization study

Decheng Meng^1^, Xin Zhang^1^, Wenfei Yu^1^, Guoliang Yin^1^, Suwen Chen^1^, Hongshuai Liu^2^, Linya Wang^2^, Fengxia Zhang^3*^

*** Correspondence:** Fengxia Zhang: fxzhang0987@163.com

## Supplementary Figures


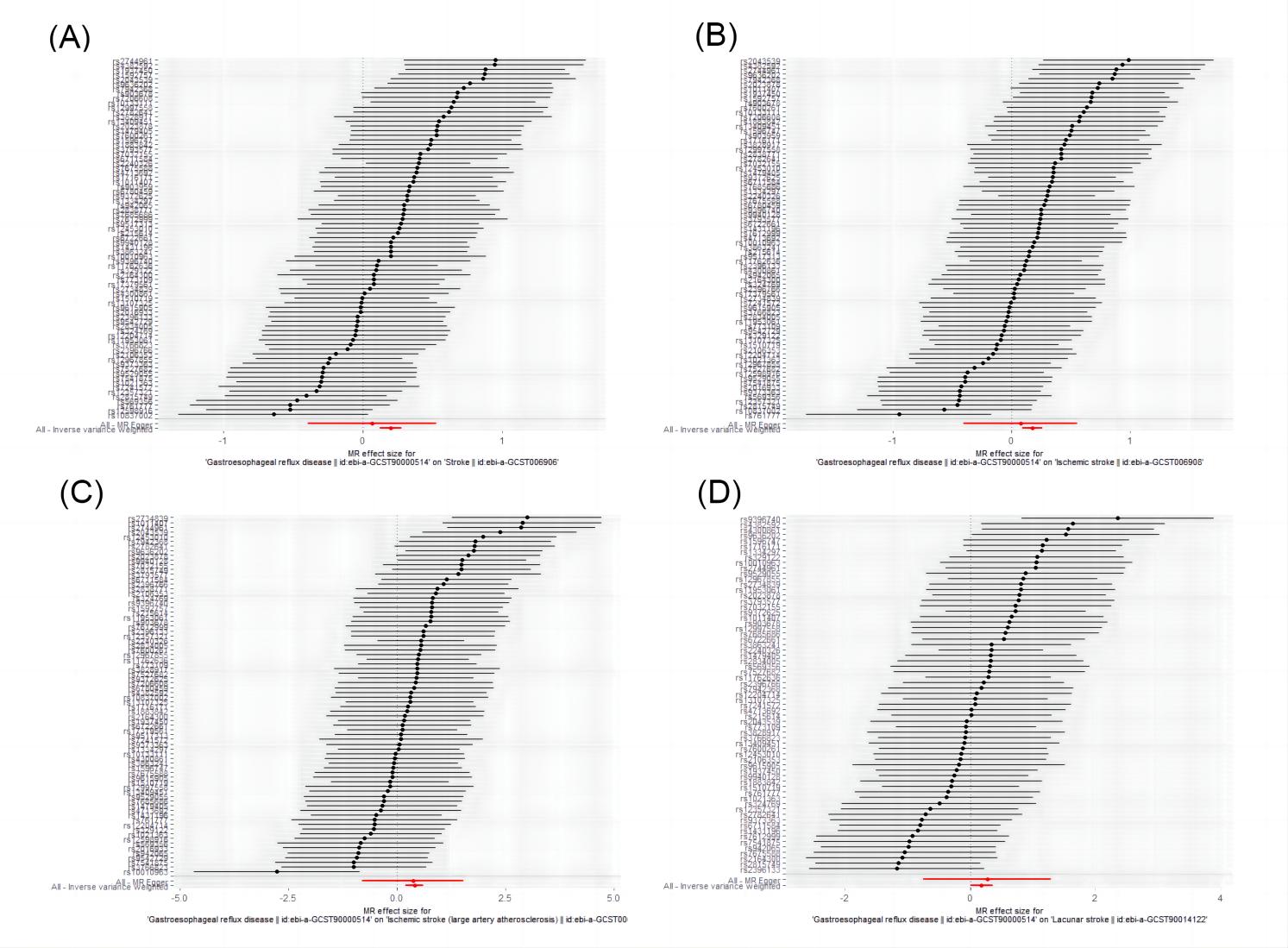


**Supplementary Figure 1.** Forest plot of genetic association between GERD and overall stroke, IS, LVS, LS.  (A) Stroke; (B) ischemic stroke; (C) large vessel stroke; (D) lacunar stroke.


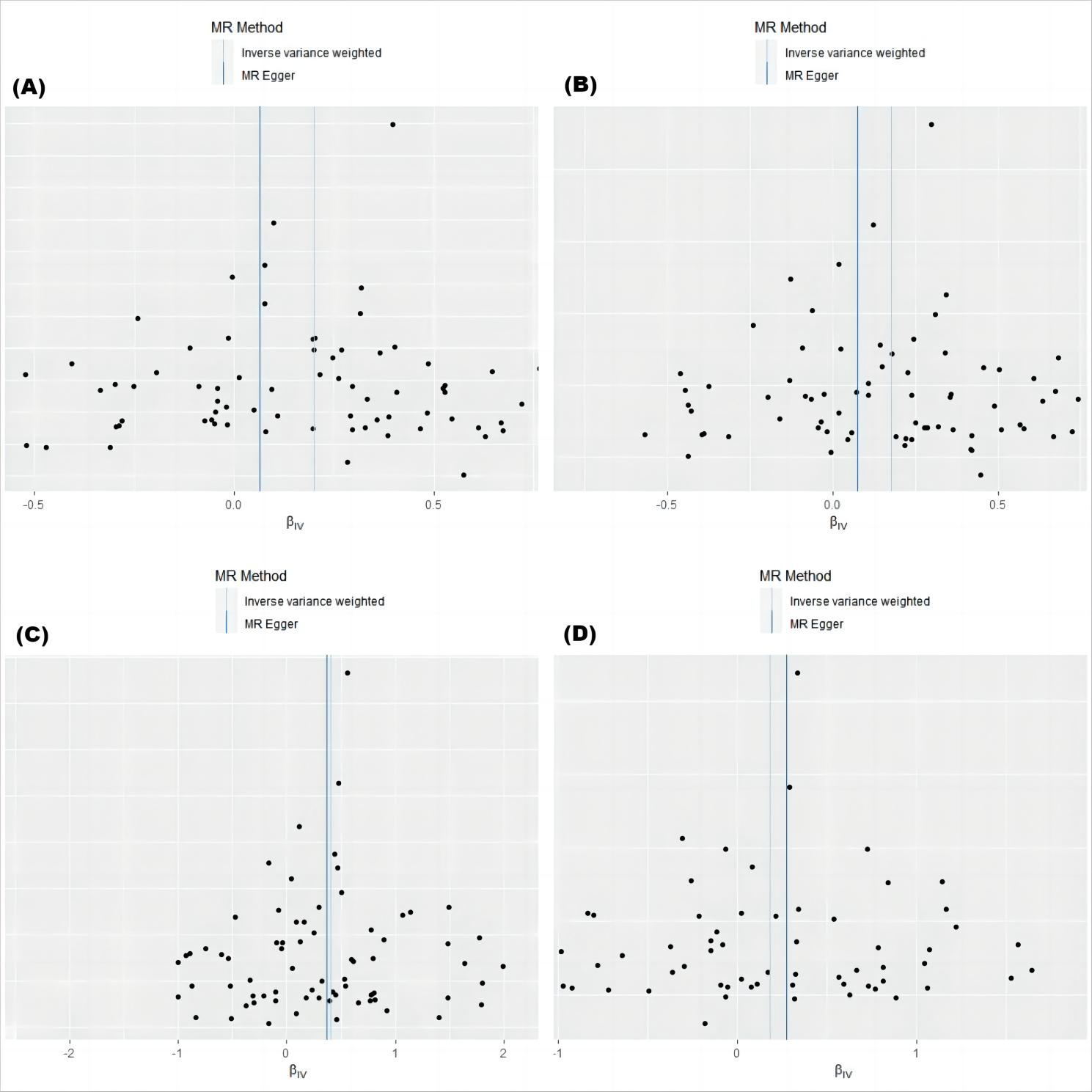


**Supplementary Figure 2.** Funnel plot of genetic association between GERD and overall stroke, IS, LVS, LS.  (A) Stroke; (B) ischemic stroke; (C) large vessel stroke; (D) lacunar stroke; Scattering points represented the effect estimated using a single SNP as an IV. The vertical lines denoted the overall estimate obtained by the IVW estimate and the MR-Egger regression.


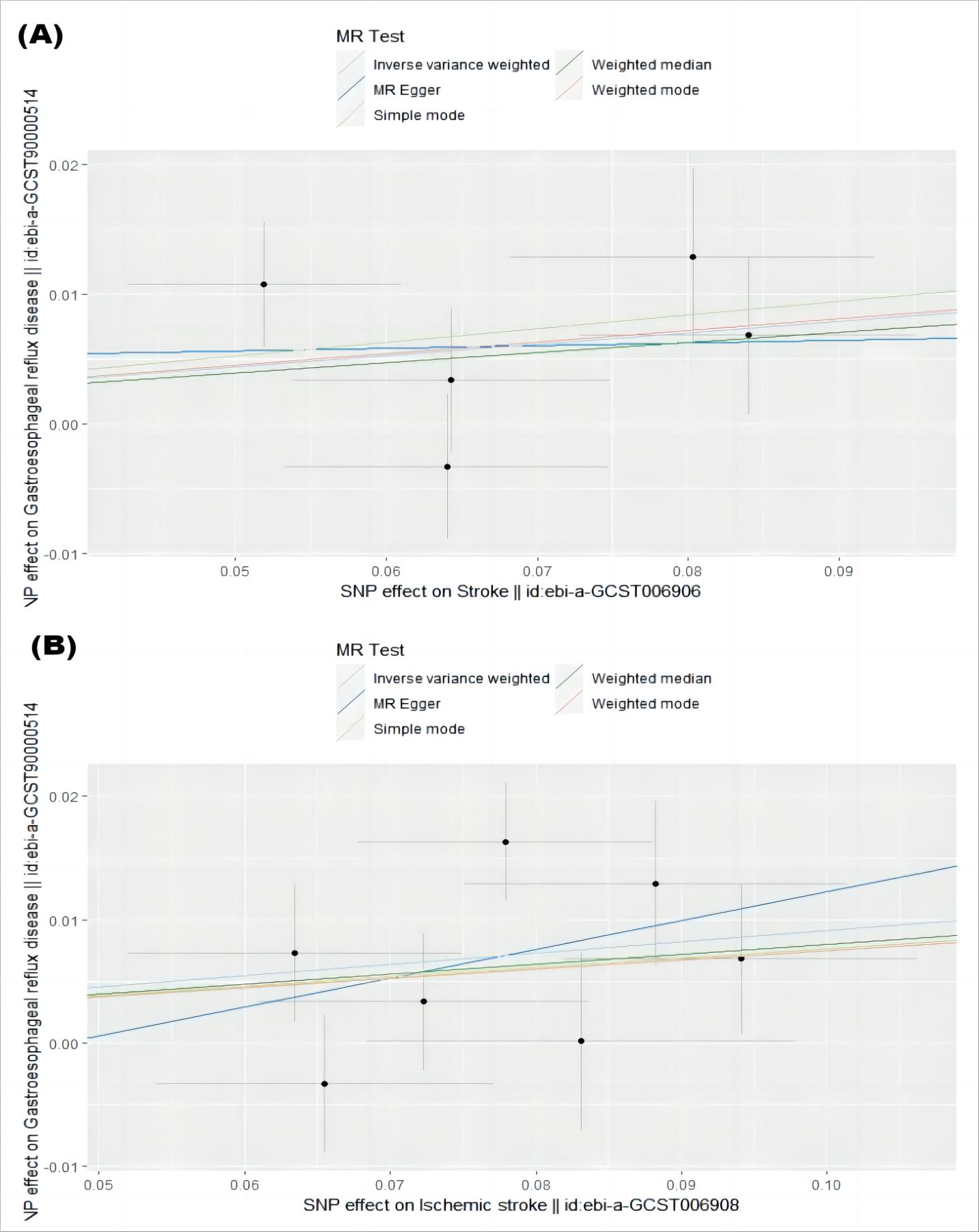


**Supplementary Figure 3.** Scatter plot of genetic association between overall stroke, IS and GERD.  (A) Stroke; (B) ischemic stroke.


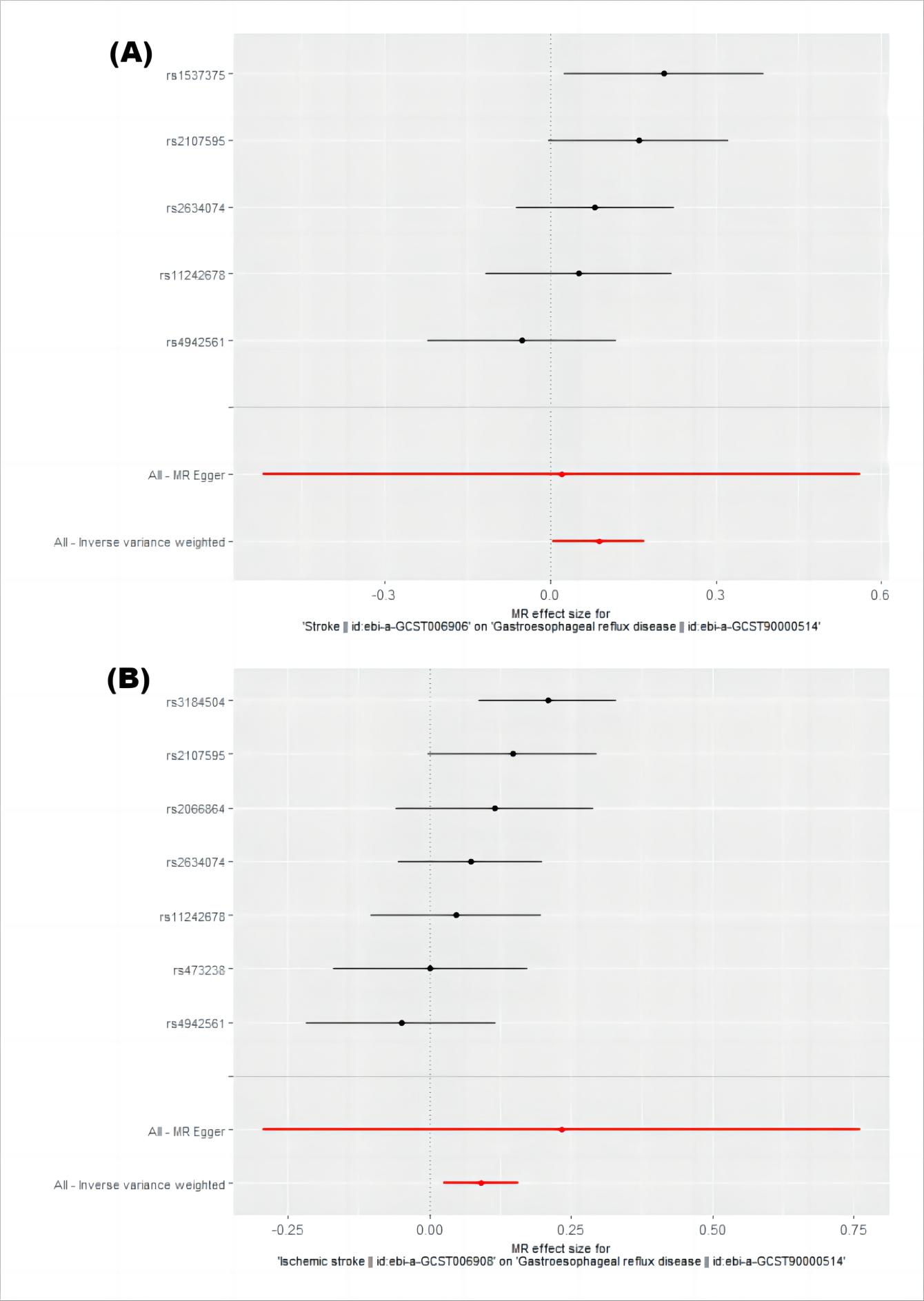


**Supplementary Figure 4.** Forest plot of genetic association between overall stroke, IS and GERD.  (A) Stroke; (B) ischemic stroke.

**
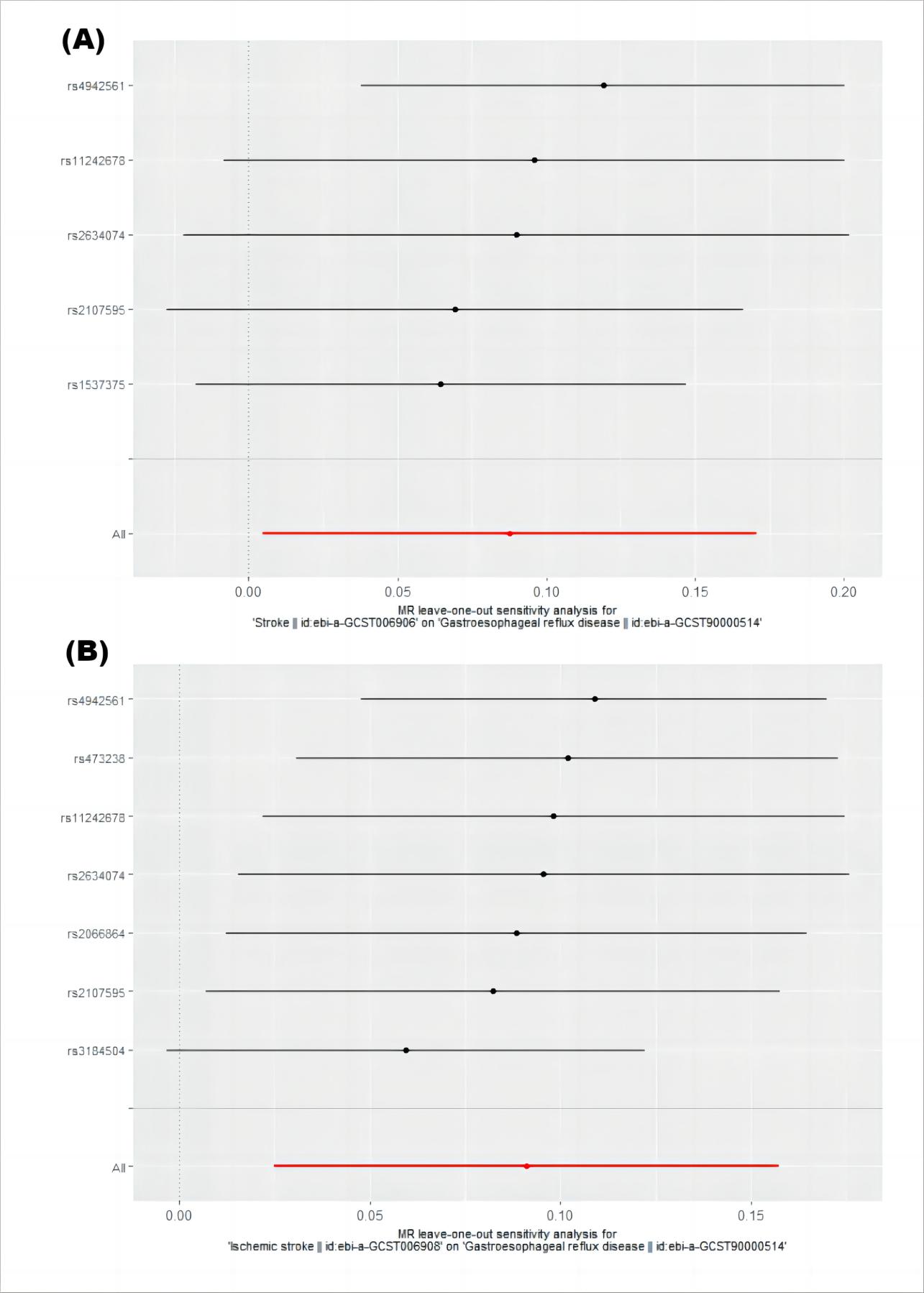
**

**Supplementary Figure 5.** The leave-one-out-sensitivity forest plot of genetic association between overall stroke, IS and GERD.  (A) Stroke; (B) ischemic stroke.
